# Feeding beta adrenergic agonists alters mitochondria metabolism in porcine skeletal muscle

**DOI:** 10.1093/jas/skaf401

**Published:** 2025-11-19

**Authors:** Con-Ning Yen, Jocelyn S Bodmer, Samuel D Gerrard, Jordan C Wicks, Morgan D Zumbaugh, Tracy L Scheffler, Samer W El-Kadi, Tim H Shi, David E Gerrard

**Affiliations:** School of Animal Sciences, Virginia Tech, Blacksburg, VA 24601; School of Animal Sciences, Virginia Tech, Blacksburg, VA 24601; School of Animal Sciences, Virginia Tech, Blacksburg, VA 24601; Department of Animal Science, University of Nebraska, Lincoln, NE 68588; Department of Animal Sciences and Industry, University of Kansas, Kansas, KS 66506; Department of Animal Sciences, University of Florida, Gainesville, FL 32611; School of Animal Sciences, Virginia Tech, Blacksburg, VA 24601; School of Animal Sciences, Virginia Tech, Blacksburg, VA 24601; School of Animal Sciences, Virginia Tech, Blacksburg, VA 24601

**Keywords:** beta agonist, energy metabolism, mitochondria, pigs, skeletal muscle

## Abstract

During skeletal muscle growth, metabolic processes regulating muscle tissue exhibit remarkable adaptability. The objective of this study was to determine the involvement of mitochondria function in the shift of metabolism in porcine skeletal muscle. To alter metabolism, we utilized β-adrenergic agonists (BAA) supplementation known to increase the proportion of fast-twitch fibers. To assess the role of the mitochondria in this process, we utilized a naturally occurring mutation in domestic pigs containing a constitutively active adenosine monophosphate activated protein kinase (AMPKγ3^R200Q^) that results in greater tissue oxidative capacity. Mature pigs with or without AMPK mutation (control and AMPKγ3^R200Q^) were fed BAA (0 and 9 ppm) for 1 wk, then were euthanized and *longissimus lumborum* muscle samples were collected and mitochondria were isolated. Mitochondria from muscle of AMPKγ3^R200Q^ pigs had higher (*P *< 0.05) oxygen consumption rates (OCR) than that of control pigs when using pyruvate/malate substrates under ADP-stimulated conditions. When provided succinate/rotenone substrates, an interaction (*P *< 0.05) was noted for basal respiration, where mitochondria from muscle of control pigs fed 0 ppm BAA had lower OCR compared to that of AMPKγ3^R200Q^ pigs and that of those fed 9 ppm BAA. These data show that BAA have more pronounced effects on control pigs than AMPKγ3^R200Q^ pigs which may be due to the inherently greater oxidative capacity of mutant pig muscle. After 1 wk of feeding BAA, there was an increase in β1-adrenergic receptor gene expression in pigs fed BAA (Treatment, *P *= 0.06; Interaction *P *= 0.08). Oxidative protein abundance increased for succinate dehydrogenase (*P *< 0.01) and citrate synthase (CS, *P *< 0.001) in AMPKγ3^R200Q^ muscle. Additionally, CS activity in isolated mitochondria from muscle of AMPKγ3^R200Q^ pigs was lower (*P *= 0.08), but whole muscle of AMPKγ3^R200Q^ pigs had overall higher CS activity (*P *< 0.01). There were no differences in glycolytic enzyme protein abundances, however, there was increased lactate dehydrogenase (*P *< 0.001) activity in muscle of control pigs and that of muscle from pigs fed BAA (*P *< 0.05). Together, these data indicate that mitochondria function is altered in porcine skeletal muscle when pigs are supplemented with BAA and suggest part of the mechanism by which BAA supplementation augments muscle growth in pigs potentially lies within the regulation of β1-adrenergic receptors and changes in mitochondrial function.

## Teaser

Skeletal muscle mitochondria function can be altered in pigs supplemented with a low dose of beta agonist. The molecular mechanism of this pathway may involve stimulation of β1-adrenergic receptor in skeletal muscle.

## Introduction

Animal growth rate and feed efficiency have drastically improved over the past 20 years in the swine industry (Wu et al., 2017). In the process of achieving such gains, changes in lean muscle mass and metabolism have been documented (Rahelic and Puac, 1981; Lefaucheur et al., 2011). Specifically, increased selection pressure on feed efficiency has resulted in the increased proportion of white, glycolytic, and fast-contracting muscle fibers (Karlsson et al., 1993; Depreux et al., 2000). These observations raise the question of whether improvements in muscle composition and mass are driven by changes in muscle energy metabolism or vice versa. Indeed, skeletal muscle function and growth alter energy metabolism through the relative contributions of glycolysis or oxidative phosphorylation responsible for creating energy substrates in muscle tissue (Glancy and Balaban, 2021). Mitochondria are a major cellular component of oxidative metabolism and contribute greatly to energy metabolism and cellular signaling in skeletal muscle tissue (Glancy and Balaban, 2021). Therefore, studying the role of the mitochondria during a shift of metabolism may provide insight into the potential mechanisms driving production efficiencies in growing meat animals.

Beta adrenergic agonists (BAA) bind the beta-adrenoceptors on muscle cells, which are coupled to a G-protein to produce cyclic adenosine monophosphate (cAMP) subsequently activating downstream signaling pathways that stimulate muscle growth (Smith, 1998; Mills et al., 2003a; Lynch and Ryall, 2008). BAA supplementation alters energy metabolism through increasing glycogenolysis and lipolysis (Maltin et al., 1990; Mills et al., 2003b). Traditionally, the BAA ractopamine, commercially available as Paylean, has been exploited in the meat industry to repartition nutrients away from adipose tissue and towards lean accretion, producing heavier, leaner carcasses with greater growth efficiencies (Beermann et al., 1987; Watkins et al., 1990; Gunawan et al., 2020). As a result of this feeding, or in the process of increasing growth efficiency, ractopamine reduces oxidative type IIA gene expression of porcine muscle (Gunawan et al., 2007) and induces a transition of oxidative type I muscle fibers to more glycolytic type II fibers in bovine skeletal muscle (Gonzalez et al., 2009). Additionally, BAA induce muscle hypertrophy through the increase of cross-sectional area and a corresponding increase in type II-specific gene expressions in muscle (Beermann et al., 1987; Zeman et al., 1988; Depreux et al., 2002; Gunawan et al., 2007). Collectively, these data indicate that feeding BAA alters the composition of skeletal muscle fiber type, enhancing energy metabolism for improved growth efficiency by discriminating against energy metabolism in support of mitochondrial function.

Pigs possessing the naturally occurring mutation that result in a constitutively active adenosine monophosphate activated protein kinase (AMPKγ3^R200Q^) have muscle with greater oxidative capacity in more traditionally considered glycolytic muscles compared to pigs without this mutation (Scheffler et al., 2011; Scheffler et al., 2014). In particular, pigs carrying the AMPKγ3^R200Q^ mutation have increased oxidative muscle fiber types containing the myosin heavy chain (MyHC) isoforms IIA and(or) IIX(D), greater mitochondria content and increased mitochondrial-based enzyme activities (Garcia-Roves et al., 2008; Park et al., 2009; Scheffler et al., 2011). Studies outlining the response of red and white muscles to BAA feeding (Gunawan et al., 2007, 2020) show a direct impact of BAA on mitochondria function but these studies have not been independent of muscle type. In this study, we exploited the oxidative nature of skeletal muscle from AMPKγ3^R200Q^ pigs to determine whether changes in mitochondria function occur during metabolic shifts caused by the feeding of BAA.

## Methods and Materials

### Ethics statement

The experimental protocol used in this study was approved by the Virginia Tech Institutional Animal Care and Use Committee (#16-199). All twenty-four pigs were raised at the Virginia Tech Swine Center in Blacksburg, VA.

### Experimental design

Pigs were genotyped for the AMPKγ3^R200Q^ mutation following Scheffler et al. (2014) procedures by isolating DNA (Zymo Research, Irvine, CA) and amplifying the area of the point change mutation with the primers 5′-AAATGTGCAGACAAGGATCTC-3′ (forward) and 5′-CCCACGAAGCTCTGCTT-3′ (reverse). Polymerase chain reaction products were digested with the restriction enzyme BsrBI (New England Biolabs, Ipswich, MA). Pigs homozygous wildtype at the AMPK loci were considered controls, while pigs that were heterozygous at the AMPK loci were considered AMPK mutants. A total of 24 pigs, with or without the AMPKγ3^R200Q^ mutation (*n* = 12 per genotype), were identified for the study. Pigs within genotype were randomly assigned to receive either 0 or 9 ppm beta adrenergic agonist (BAA, ractopamine hydrochloride, DAC, Dover, OH, *n* = 6 per genotype) meeting current USDA guidelines for administration for 1 wk. Pigs were individually housed in pens to record feed intake and selected pigs were administered (top dressed) dietary BAA at a rate of 9 ppm. All pigs were fed ad libitum with a finisher diet formulated to meet al. nutrient requirements established by NRC (2012) and compatible with BAA feed additive directions (Table 1). After receiving 1 wk of feed additives, pigs (approximately 140 kg) were harvested at the Virginia Tech Meat Teaching, Extension and Research Center following standard procedures and in accordance with Virginia State Inspection Regulations. At harvest, *longissimus lumborum* (LL) samples were collected within 5 min of exsanguination. Muscle samples were either snap frozen in liquid nitrogen or used for fresh mitochondrial isolation.

**Table 1.** Ingredient and nutrient composition of swine finisher diet

Ingredients	Percentage (%)
**Yellow corn**	77.8
**Soybean meal**	17.7
**Soy oil**	2
**Dicalcium phosphate**	0.6
**Limestone**	0.8
**Salt**	0.2
**L-Lysine**	0.2
**Vitamin and mineral mix^1^**	0.125
**L-Threonine**	0.1
**DL-Methionine**	0.06
**L-Tryptophan**	0.02
**Phytase**	0.02
**Calculated analysis**	
**Metabolizable energy, kcal/kg**	3,356
**Protein, %**	16.01
**Fat, %**	4.89
**Calcium, %**	0.47
**Phosphorus, %**	0.43
**Animo acid analysis, W/W%**	
**Leucine**	1.37
**Lysine**	0.95
**Arginine**	0.91
**Valine**	0.74
**Phenylalanine**	0.73
**Isoleucine**	0.65
**Threonine**	0.62
**Histidine**	0.40
**Methionine**	0.30
**Tryptophan**	0.20

1 Vitamin and Mineral Mix provided per kilogram of diet: 187,000 mg of calcium, 139,000 mg of phosphorus, 17,000 mg of potassium, 17,000 mg of magnesium, 10,000 mg of iron, 20 mg of cobalt, 1,200 mg of copper, 135 mg of iodine, 3,200 mg of manganese, 15 mg of selenium, 4,800 mg of zinc, 660,793 IU of vitamin A, 66,079.3 IU of vitamin D3, 2,202.6 IU of vitamin E, 1.3 mg of vitamin B12, 220.3 mg of menadione, 440.5 mg of riboflavin, 1,530.8 mg of D-pantothenic acid, 2,202.6 mg of niacin, and 24,229.1 mg of choline.

### Immunoblotting

Frozen muscle samples were ground to a fine powder in liquid nitrogen with a mortar and pestle and aliquoted and stored at −80 °C. SDS-PAGE samples were processed following Zeng et al. (2022) with minor modifications. Briefly, 100 mg of tissue was homogenized in RIPA buffer (50 mM tris-hydrochloride, 150 mM sodium chloride, 1% NP-40, 0.25% sodium deoxycholate, protease inhibitor, and phosphatase inhibitor) using a Polytron homogenizer (Kinematica, Malters, Switzerland). Homogenized tissue lysates were centrifuged at 13,000 × g for 5 min at 4 °C. Resulting supernatants were collected to determine protein concentration with bicinchoninic acid protein assay kit (Pierce, Rockford, IL). Protein concentrations were diluted to 3 mg/mL with sample buffer (0.25 M tris-hydrochloride, 0.5 M dithiothreitol, 10% sodium dodecyl sulfate, 0.5% bromophenol blue, 50% glycerol). Equal amounts of sample were loaded to achieve 18 µg of protein per well and subjected to SDS-PAGE and transferred to nitrocellulose membranes following that reported by Yen et al. (2024). Total protein was determined with the addition of Ponceau S (0.1% ponceau s and 5% acetic acid) and imaged with ChemiDoc XRS+ imaging system (Bio-Rad, Hercules, CA). Membranes were then washed with 1X TBS (20 mM tris base and 140 mM sodium chloride) until Ponceau S stain was removed. Membranes were blocked in 5% non-fat milk solution diluted in 1X TBST (20 mM tris base, 140 mM sodium chloride, and 0.1% Tween) for 1 h at 25 °C. The following primary antibodies were incubated overnight: lactate dehydrogenase subunit A (LDHA, Novus Biologicals 48336, Centennial, CO, USA), glyceraldehyde 3-phosphate dehydrogenase (GAPDH, Novus Biologicals 300-221, Centennial, CO, USA), succinate dehydrogenase (SDHA, Abcam 14715, Cambridge, UK), citrate synthase (CS, Santa Cruz Biotechnology 390693, Dallas, TX, USA), voltage-dependant anion channel (VDAC, Cell Signaling Technology 4461, Danvers, MA, USA). Membranes were washed three times with 1X TBST for 5 min before secondary antibodies (LI-COR, Lincoln, NE) were added for 1 h at 25 °C. Membranes were scanned with Odyssey Scanner (LI-COR, Lincoln, NE). Images were quantified using Image Studio Lite 5.2 (LI-COR, Lincoln, NE). All target proteins were normalized to total protein from Ponceau S-stained blots.

### Enzyme activity

Oxidative citrate synthase enzyme activity was assessed following procedures reported by Scheffler et al. (2014). Briefly, 100 mg of frozen tissue was diluted 1:10 with reaction buffer (100 mM tris, 1 mM DTNB (5,5′-dithiobis-[2-nitrobenzoic acid]) and 10 mM oxaloacetate) and homogenized with a Polytron homogenizer (Kinematica, Malters, Switzerland). Alternatively, isolated mitochondria were normalized to protein concentration and sonicated with a S-4000 sonicator (QSonica, Newton, CT) and diluted at 1:40. Citrate synthase activity was determined by measuring the reduction of DTNB at 412 nm using a microplate spectrophotometer (Biotek, Winooski, VT). Glycolytic lactate dehydrogenase activity was assessed following Wright et al. (2018). Briefly, 100 mg of frozen tissue was diluted 1:10 with reaction buffer (20 mM tris base, 40 mM potassium chloride, 1.3 mM EGTA (ethylene glycol-bis(β-aminoethyl ether)-N, N, N′,N′-tetraacetic acid), 50 mM sucrose) and homogenized with Polytron homogenizer (Kinematica, Malters, Switzerland). Lactate dehydrogenase activity was determined by measuring the oxidation of NADH to NAD at 340 nm using a microplate spectrophotometer (Biotek, Winooski, VT). Both enzyme activity samples were normalized to total protein determined with bicinchoninic acid protein assay kit (Pierce, Rockford, IL) and final units are reported as µmol/min/mg protein.

### Mitochondrial respiration

Briefly, mitochondria were isolated from LL muscle through differential centrifugation following Scheffler et al. (2015). Final isolated mitochondria protein concentration was determined with bicinchoninic acid protein assay kit (Pierce, Rockford, IL) and samples were diluted to 1 µg/µL with mannitol sucrose medium (220 mM mannitol, 70 mM sucrose, 10 mM Tris-HCl, and 1 mM EGTA, pH 7.4). Mitochondrial respiration was determined using Seahorse XFe96 (Agilent, Santa Clara, CA) following Yen et al. (2024). The following substrates were utilized to assess mitochondria respiration capacity: 10 mM pyruvate and 5 mM malate (PM), 10 mM succinate and 2 µM rotenone (SR), 10 mM glutamate and 5 mM malate (GM), and 40 µM palmitoyl-carnitine and 1 mM malate (PCM). Diluted fresh mitochondria were loaded on the Seahorse plate at the following concentrations per well: 1.5 µg for PM, 1 µg for SR, 2 µg for GM, and 1.5 µg for PCM. Mitochondrial oxygen consumption rate (OCR) was measured in a series of injections to target electron transport chain function. Baseline represents basal respiration of isolated mitochondria with substrates. To assess oxidative phosphorylation (OXPHOS) capacity 4 mM ADP (Sigma-Aldrich, Darmstadt, Germany) was added to determine ADP-stimulated respiration. Proton leak was determined with 2 µM oligomycin (Tocris Bioscience, Bristol, UK). The uncoupler carbonyl cyanide 4 (trifluoromethoxy) phenylhydrazone (FCCP; Sigma-Aldrich, Darmstadt, Germany) was added to a final concentration of 4 µM to measure for maximal respiration. Non-mitochondrial respiration was determined with the 4 µM antimycin A (Sigma-Aldrich, Darmstadt, Germany). All respiration data were normalized to the µg of isolated mitochondria protein loaded per well and data are displayed only as mitochondrial oxygen consumption, which is determined by subtracting non-mitochondrial respiration from all injections.

### Gene expression

RNA was isolated from 50 mg of frozen ground tissue using Quick-RNA prep kit (Zymo Research, Irvine, CA). Quantity and quality of RNA were measured with NanoDrop (Fisher Scientific, Waltham, MA) and diluted to 60 ng/µL to generate complementary DNA with reverse transcriptase (Applied Biosystems, Foster City, CA). The following genes (Table 2) were assessed: *succinate dehydrogenase* (*SDHA*), *citrate synthase* (*CS*), *glycogen synthase* (*GS*), *peroxisome proliferator-activated receptor gamma coactivator 1-alpha* (*Pgclα*), *β1*- and *β2-adrenergic receptors* (*AR*). All gene expressions were normalized to ribosomal RNA (*18 s*) and displayed as a fold change of control muscle to AMPK muscle.

**Table 2.** List of primers for mRNA gene expression of the following genes

Accession number	Gene and primer name	Primer sequence (5′ to 3′)
**DQ402993.1**	Succinate dehydrogenase complex subunit A^1^	
	SDHA-Forward	ACTGGAGGTGGCATTTCTAC
	SDHA-Reverse	GCCGTAATTCTCTAGCTCTACC
**NM_213963.2**	Peroxisome proliferator-activated receptor gamma coactivator 1-alpha^1^	
	Pgc1a-Forward	GATCACGTTCAAGATCTCCCTAC
	Pgc1a- Reverse	AGACTCCCGCTTCTCATACT
**NM_214276**	Citrate synthase^2^	
	CS-Forward	TGCCAGTGCTTCTTCCACGAACTT
	CS-Reverse	GTTGCCGTGTTGCTGCCTGAA G
**AJ507152**	Glycogen synthase^2^	
	GS-Forward	CCCAGTGGGAGGAGGCAGTCT TG
	GS-Reverse	GAACCGCCGGTCCAGAATGTAGA
**AF042454.1**	β-Adrenergic receptor 1^2^	
	*β_1_*-AR-Forward	CTGCGAAGACTAGGGAAGGGATGG
	*β_1_*-AR-Reverse	CCCCGGGAACGGAATGGAA
**NM_001128436.1**	β-Adrenergic receptor 2^2^	
	*β_2_*-AR-Forward	GGCTGCCCTTCTTCATCGTCAAC
	*β_2_*-AR-Reverse	AGCCCACCCAGTTTAGCAGGATGT
**NR_046261.1**	18s ribosomal^3^	
	18s-Forward	GTAACCCGTTGAACCCCAT
	18s-Reverse	CCATCCAATCGGTAGTAGCG

1 Scheffler et al. (2014).

2 Gunawan et al. (2007).

3 Chen et al. (2017).

### Mitochondrial DNA abundance

DNA was isolated from 50 mg of frozen powered muscle using Quick DNA Prep Kit (Zymo Research, Irvine, CA). mtDNA was quantified using real-time polymerase chain reactions as described by López-Andreo et al. (2005). Relative quantity of mtDNA was calculated by comparing fold change of control muscle to AMPK muscle.

### Statistics

Prior to data analysis, normality distribution was tested with the Shapiro–Wilk test. Data were analyzed as a complete randomized design in a 2 × 2 factorial arrangement, considering AMPKγ3^R200Q^ genotype, BAA treatment, and their interaction as fixed effects. Harvest date was considered a random effect and animals served as experimental units. Least square means and standard error bars were obtained with SAS Proc Mixed procedure. Statistical significance is denoted as *P < 0.05, **P < 0.01, ***P < 0.001 or unless otherwise stated. Post hoc analysis was determined using Tukey HSD with significance level of *P* < 0.05 or unless otherwise stated. Significantly different main effects of AMPKγ3^R200Q^ genotype (Control and AMPK mutant) and β-adrenergic agonists (BAA) treatment (0 ppm and 9 ppm) are displayed when there is no difference of interaction between main effects.

## Results

**Figure 1.**
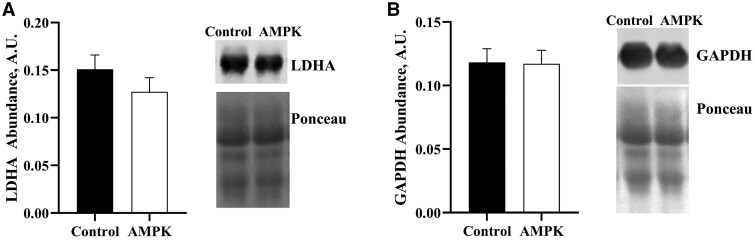
Effect of AMPKγ3^R200Q^ genotype (Control and AMPK mutant) on glycolytic protein abundance from *longissimus lumborum* (LL) muscle. Glycolytic enzyme protein abundance of lactate dehydrogenase (LDHA; A) and glyceraldehyde 3-phosphate dehydrogenase (GAPDH, B). Representative western blot images of genotype without BAA treatment samples that were loaded equally to obtain 18 µg of total protein for LDHA, GAPDH, and total protein stain (Ponceau). All values are displayed as least square means followed by standard error bars.

**Figure 2.**
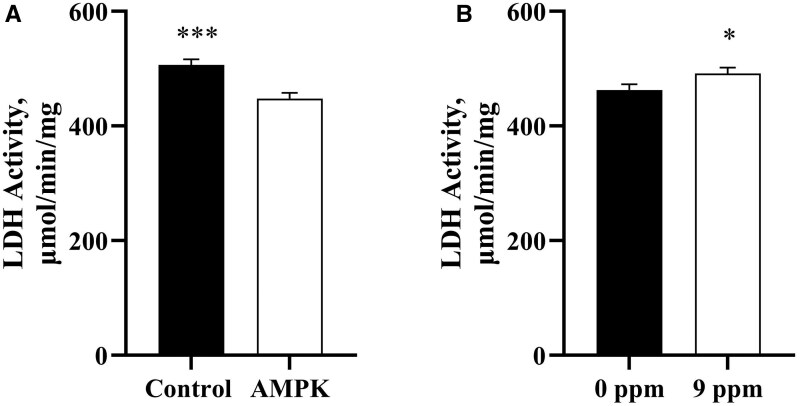
(A) Effect of AMPKγ3^R200Q^ genotype (Control and AMPK mutant) on lactate dehydrogenase activity (LDH, µmol/min/mg) from *longissimus lumborum* (LL) muscle. (B) Effect of β-adrenergic agonists (0 ppm or 9 ppm BAA) on LDH activity. All values are displayed as least square means followed by standard error bars. Significance is denoted as *P < 0.05 and ***P < 0.001.

After feeding 1 wk of β-adrenergic agonists (BAA) there was increased final body weight in pigs fed BAA but no differences of main effects or their interaction on feed: gain or average daily gain (Table 3). There were no differences in glycolytic protein abundances of lactate dehydrogenase (LDHA) and glyceraldehyde 3-phosphate dehydrogenase (GAPDH) across the main effects of genotype or treatment in *longissimus lumborum* (LL) muscle of pigs (Figure 1). However, when assessing enzyme activity there were genotype and treatment differences in lactate dehydrogenase (LDH) activity (Figure 2). While there were no differences in LDHA abundance between genotypes (Figure 1A), there was greater (*P *< 0.001) LDH activity in control muscle compared to the AMPKγ3^R200Q^ mutated pig muscle (Figure 2A). Additionally, pigs supplemented with 9 ppm BAA yielded greater (*P *< 0.05) muscle LDH activity than that of pigs fed 0 ppm BAA (Figure 2B).

**Table 3.** Effect of AMPKγ3^R200Q^ genotype (Control and AMPK mutant) and β-adrenergic agonists (0 or 9 ppm BAA) on pig performance

	Control		AMPK mutant		*P*-Value		
	0 ppm	9 ppm	0 ppm	9 ppm	Genotype	Treatment	Interaction
**Initial weight, kg**	120.61 ± 6.54	131.05 ± 6.54	120.2 ± 6.54	132.22 ± 6.54	0.95	0.10	0.91
**Slaughter weight, kg**	135.44 ± 6.32^b^	143.29 ± 6.32^a^	126.0 ± 6.32^b^	145.91 ± 6.32^a^	0.58	0.03	0.34
**Feed: gain**	3.01 ± 0.64	3.34 ± 0.64	3.76 ± 0.64	3.64 ± 0.64	0.43	0.88	0.73
**ADG, kg/d**	1.66 ± 0.30	1.75 ± 0.30	1.39 ± 0.30	1.36 ± 0.30	0.31	0.92	0.84

**Figure 3.**
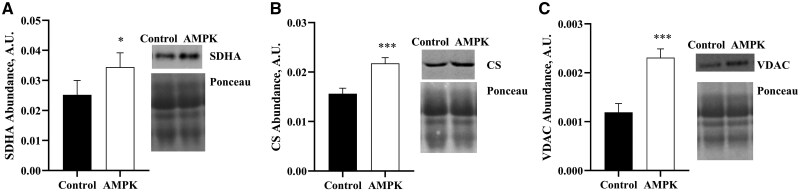
Effect of AMPKγ3^R200Q^ genotype (Control and AMPK mutant) on oxidative protein abundance from *longissimus lumborum* (LL) muscle. Mitochondrial protein abundance of succinate dehydrogenase (SDHA; A), citrate synthase (CS; B), and voltage-dependent anion channel (VDAC; C). Representative western blot images of genotype without BAA treatment samples for that were loaded equally to obtain 18 µg of total protein for SDHA, CS, VDAC, and total protein stain (Ponceau). All values are displayed as least square means followed by standard error bars. Significance is denoted as *P < 0.05 and ***P < 0.001.

**Figure 4.**
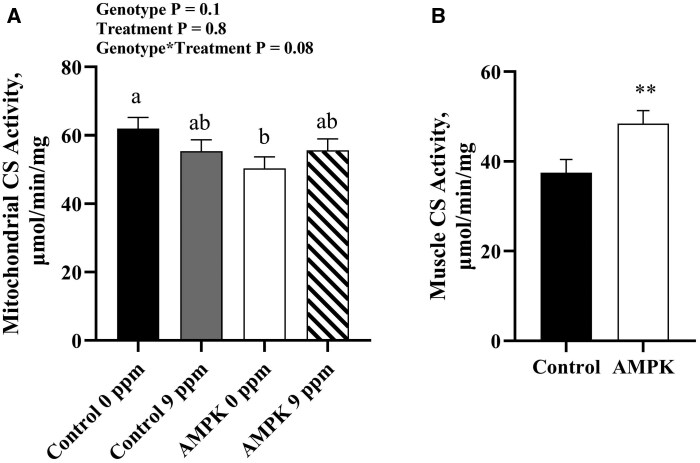
(A) Interaction of AMPKγ3^R200Q^ genotype (Control and AMPK mutant) and β-adrenergic agonists (0 ppm or 9 ppm BAA) on citrate synthase activity (CS, µmol/min/mg) from isolated mitochondria. (B) Effect of AMPKγ3^R200Q^ genotype (Control and AMPK mutant) on citrate synthase activity (CS, µmol/min/mg) from *longissimus lumborum* (LL) muscle. All values are displayed as least square means followed by standard error bars. Significance is denoted as **P < 0.01 for main effects and *P* = 0.08 for interaction.

Greater amounts of oxidative enzyme proteins were detected (*P *< 0.05) in muscle of AMPKγ3^R200Q^ pigs compared to that of control pigs and there were no statistically significant differences in treatment main effect nor their interaction (Figure 3). AMPKγ3^R200Q^ pig muscle contained greater amounts of succinate dehydrogenase (SDHA, *P *< 0.05, Figure 3A), citrate synthase (CS, *P *< 0.001, Figure 3B), and voltage-dependent anion channel (VDAC, *P *< 0.001, Figure 3C). Even though there was a greater abundance of CS in AMPKγ3^R200Q^ muscle (Figure 3B), when CS enzyme activity was determined in isolated mitochondria, a genotype by treatment interaction was noted (*P *= 0.08) where muscle of control pigs had the greatest CS activity, followed by muscle from control and AMPKγ3^R200Q^ pigs fed 9 ppm BAA, and muscle of AMPKγ3^R200Q^ mutant pigs fed 0 ppm BAA had the least CS activity (Figure 4A). However, when evaluating CS enzyme activity on a per mg of muscle protein basis, the AMPKγ3^R200Q^ muscle contained greater (*P *= 0.01) CS activity compared to that of control pigs (Figure 4B).

**Figure 5.**
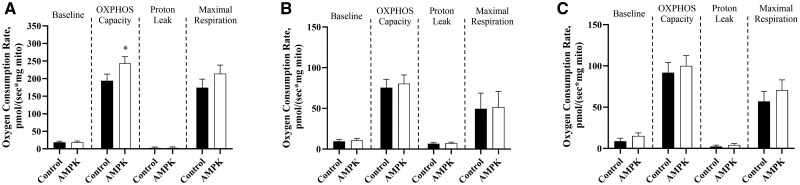
Effect of AMPKγ3^R200Q^ genotype (Control and AMPK mutant) on mitochondria oxygen consumption rate of isolated mitochondria from porcine *longissimus lumborum* (LL) under saturating concentrations of pyruvate/malate (A), glutamate/malate (B), and palmitoyl-carnitine/malate (C). Baseline represents basal respiration of isolated mitochondria with substrates. OXPHOS capacity is ADP (5 mM) stimulated respiration. Proton leak is determined with 2 µM oligomycin. Maximal respiration is achieved with the uncoupler FCCP (4 µM). All values are displayed as least square means followed by standard error bars. Significance is denoted as *P < 0.05.

**Figure 6.**
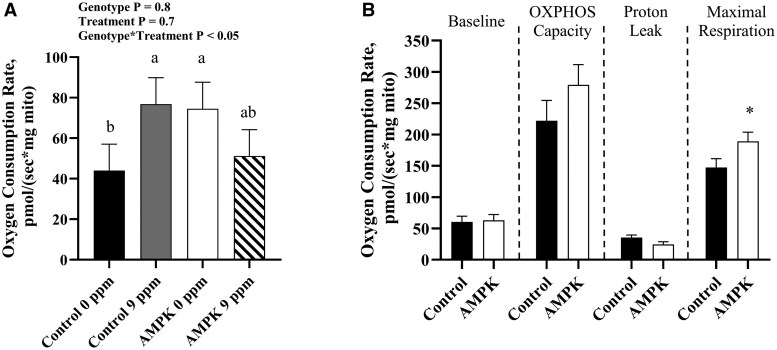
(A) Interaction of AMPKγ3^R200Q^ genotype (Control and AMPK mutant) and β-adrenergic agonists (0 ppm or 9 ppm BAA) on baseline mitochondria oxygen consumption rate under saturating concentrations of succinate/rotenone. (B) Effect of AMPKγ3^R200Q^ genotype (Control and AMPK mutant) on mitochondria oxygen consumption rate of isolated mitochondria from porcine *longissimus lumborum* (LL) under saturating concentrations of succinate/rotenone. Baseline represents basal respiration of isolated mitochondria with substrates. OXPHOS capacity is ADP (5 mM) stimulated respiration. Proton leak is determined with 2 µM oligomycin. Maximal respiration is achieved with the uncoupler FCCP (4 µM). All values are displayed as least square means followed by standard error bars. Significance is denoted as *P < 0.05.

Regarding mitochondrial function, AMPKγ3^R200Q^ mutant mitochondria had greater (*P *< 0.05) OXPHOS capacity than that of their control counterparts when provided pyruvate/malate substrates (Figure 5A). No genotype or BAA treatment main effects were noted with glutamate/malate (Figure 5B) or palmitoyl-carnitine/malate (Figure 5C) as oxidative substrates. However, when provided succinate/rotenone there was a genotype by BAA treatment interaction (*P *< 0.05) in baseline respiration where mitochondria from muscle of control pigs fed 0 ppm BAA possessed the least oxygen consumption rate (OCR), while those from muscle of AMPKγ3^R200Q^ mutant pigs fed 9 ppm BAA had intermediate OCR, and the greatest OCR were of mitochondria isolated from control pigs fed 9 ppm BAA and AMPKγ3^R200Q^ mutant pigs fed 0 ppm BAA (Figure 6A). Additionally, there was a main effect of genotype where AMPKγ3^R200Q^ mutant mitochondria had greater (*P *< 0.05) maximal respiration than control mitochondria (Figure 6B).

**Figure 7.**
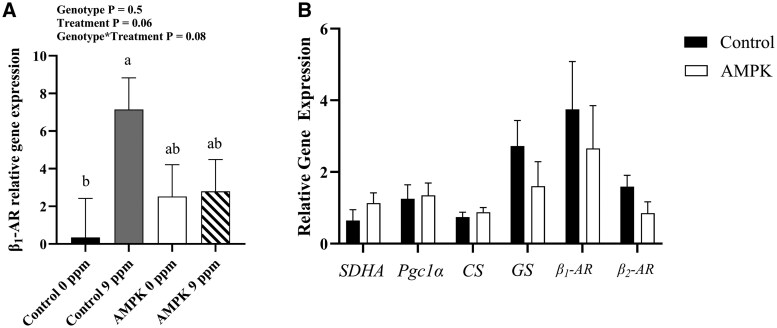
Gene expression (fold change) from *longissimus lumborum* (LL) muscle. (A) Interaction of AMPKγ3^R200Q^ genotype (Control and AMPK mutant) and β-adrenergic agonists (0 ppm or 9 ppm BAA) on *β_1_-AR* gene expression. (B) Effect of AMPKγ3^R200Q^ genotype (Control and AMPK mutant) on gene expression of *succinate dehydrogenase* (*SDHA*), *peroxisome proliferator-activated receptor gamma coactivator 1-alpha* (*Pgc1a*), *citrate synthase* (*CS*), *glycogen synthase* (*GS*), *β_1_- and β_2_-adrenergic receptors* (*AR*). All values are displayed as least square means followed by standard error bars. Significance is denoted as *P* < 0.08 for main effect and interaction.

Gene expression of key metabolic regulators of BAA were determined through quantitative polymerase chain reaction. *β_1_-adrenergic receptor* (*β_1_-AR*) gene expression was greater (*P *= 0.06) in muscle of pigs fed 9 ppm BAA than that of pigs fed 0 ppm BAA (Figure 7A). An interaction between genotype and treatment (*P *= 0.08) gene expression of *β_1_-AR* was detected where muscle from control pigs fed 9 pm BAA had a greatest *β_1_-AR* expression, AMPKγ3^R200Q^ mutants regardless of BAA treatment had intermediate expression, and control pigs fed 0 ppm BAA had the least expression (Figure 7A). No genotype (Figure 7B) or treatment effects were evident when probing for most muscle genes of interest. Additionally, no differences in mtDNA abundance were noted across any treatment combinations (data not shown).

## Discussion

In skeletal muscle, lactate dehydrogenase (LDH) reduces pyruvate to lactate in the absence of oxygen (Dawson et al., 1964). LDH is composed of 4 subunits in various combinations that form five isoforms specific to either skeletal or cardiac muscle. Each isoform of LDH has different affinities to substrates, inhibition concentrations, isoelectric point that can ultimately affect enzyme function (Valvona et al., 2016). LDH abundance in skeletal muscle cells is one of many indicators of glycolytic metabolism due to the presence of increased glycolytic flux and subsequent production of lactate (Spriet et al., 2000). Yet, enzyme abundance does not necessarily indicate enzyme functionality or activity because LDH activity is regulated by substrate abundance (Forkasiewicz et al., 2020). Consistent with this notion, Scheffler et al. (2016) reported no changes in lactate content (µmol/g) in the skeletal muscle of pigs harboring the AMPKγ3^R200Q^ mutation but did not address the abundance or function of LDH. In the current study, no differences in lactate dehydrogenase subunit a (LDHA) abundance were noted in the muscle of pigs with or without the AMPKγ3^R200Q^ mutation, yet enzyme assays indicate that control pigs have more LDH activity. Similarly, rodents possessing a chronically activated AMPK have a reduction of muscle LDH activity when compared to controls (Putman et al., 2003). These results could be partially due to the influence of AMPK activation on genes regulating lipid and glucose metabolism. Moreover, in transgenic mice overexpressing AMPKγ3, no changes in *ldh2* gene expression were detected; however, when AMPKγ3 was ablated, *ldh2* gene expression increased when animals were fasted suggesting that AMPK regulates the expression of glucose responsive genes (Long et al., 2005). Herein, we report no treatment differences in LDHA protein abundance, yet LDH activity was increased with BAA supplementation. Our data are consistent with observations of Kaundal and Sharma (2011) and Hoshino et al. (2012) which reported an increase in LDH activity in mouse skeletal muscle after BAA supplementation. Beta adrenergic agonist feeding alters LDH enzyme activity through the activation of beta receptors that cause an increase of cyclic adenosine monophosphate levels which then activates protein kinase A to phosphorylate LDH (Helmreich et al., 1976; Huang et al., 1995; Jungmann and Kiryukhina, 2005). Additionally, McElligott et al. (1989) reported an increase in skeletal muscle lactate produced after BAA supplementation in rats. Together these data indicate that LDH abundance is not necessarily linked to functionality, which suggests that there is an important distinction of assessing protein abundance and enzyme functionality in respect to their overall contribution to metabolism.

Citrate synthase (CS) is located in the matrix of mitochondria and is generally considered the first step of the Kreb’s cycle and its abundance is widely considered an indication of mitochondrial abundance (Srere, 1974; Williams et al., 1986). However, CS is encoded by nuclear DNA and therefore must be translated to protein in the cytoplasm and transported through both membranes of the mitochondria before arriving at its functional destination within the mitochondrial matrix (Harmey and Neupert, 1979). Because of the disparity in location, we evaluated citrate synthase (CS) activity in either an isolated mitochondria or whole muscle protein basis. Previously, we reported that sample preparation and location are important features in capturing the full complexity of mitochondria function and metabolism (Yen et al., 2024). In the current study, CS activity in isolated mitochondria resulted in a genotype by treatment interaction with an overall greater CS activity in control pigs compared to AMPKγ3^R200Q^ mutant isolated mitochondria. This suggests that on a per mg of protein of isolated mitochondria basis, CS activity is lower in pigs harboring the AMPKγ3^R200Q^ mutation. As described in Lyons et al. (2006), the amount of CS DNA and mRNA is not correlated with CS activity, as observed in rat gastrocnemius muscle which had the lowest qualities of CS mRNA expression but the greatest CS activity levels. These data could highlight the critical nature of posttranscriptional controls in CS activity. Regardless, pigs harboring a constitutively active AMPK leads to increased mitochondrial biogenesis and could potentially have posttranslational modifications on CS enzyme regulation that can alter enzyme activity. However, on a whole muscle basis, the AMPKγ3^R200Q^ mutant pigs contained greater CS activity, because there is more CS abundance on a whole muscle fraction. Lebret et al. (1999) and Scheffler et al. (2014) showed that longissimus muscle of AMPKγ3^R200Q^ mutant pigs had greater CS and beta-hydroxyl-acyl-coenzyme A dehydrogenase (HAD) enzyme activity compared to wildtype muscle. In the current study, we were unable to detect any differences in CS activity due to BAA treatment. However, given the short duration and low dosage of BAA fed, at least compared to previous studies, this is not surprising. Hoshino et al. (2012) showed that 3 wk feeding of the beta adrenergic agonist clenbuterol in mice resulted in muscle mitochondria with reduced CS and HAD activity and differences in pyruvate oxidation based on isolated mitochondria fractions. Regardless, these data show determining enzyme activity on different sample preparations results in different signals about the contributions of mitochondrial enzymes on overall metabolism. Further research will be necessary to determine the specific effect of AMPK on posttranslational modifications of enzymes of energy metabolism.

Measuring mitochondrial respiration is a means to assess the functionality of the mitochondria through oxygen consumption rate (OCR). Scheffler et al. (2014) showed that AMPKγ3^R200Q^ mutated mitochondria have greater ADP-stimulated OCR when provided substrates pyruvate/malate and succinate/rotenone. Consistent with those findings, our studies show isolated mitochondria from AMPKγ3^R200Q^ pigs have higher OCR when provided pyruvate/malate and succinate/rotenone. When provided glutamate/malate and palmitoyl-carnitine/malate no changes were observed suggesting no difference in the mitochondria’s ability to utilize the aspartate-malate shuttle or long-chain fatty acid uptake. Similarly, using muscle mitochondria from transgenic mice harboring the overexpression of AMPKγ3, no differences in mitochondrial respiration were observed between transgenic and wildtype mice when provided glutamate, pyruvate, and malate (Garcia-Roves et al., 2008). The most interesting finding in the current study was the genotype and treatment interaction for basal respiration where control isolated mitochondria from muscle of pigs fed 9 ppm BAA had the same OCR as mitochondria from AMPKγ3^R200Q^ mutant pig muscle fed 0 ppm BAA, which had greater OCR compared to control pigs fed 0 ppm BAA. First, BAA supplementation can increase mitochondrial basal respiration of control pigs, which is a similar finding to isolated mitochondria from muscle of mice supplemented with the beta adrenergic agonist isoproterenol that had increased basal respiration and maximal respiration in the presence of glutamate, malate, and succinate (Azevedo Voltarelli et al., 2021). Second, BAA supplementation did not have the same effect on pigs harboring the AMPKγ3^R200Q^ mutation, suggesting that increased oxidative capacity in skeletal muscle may have differing sensitivities to BAA supplementation. A possible explanation for why mitochondria of AMPKγ3^R200Q^ mutant pigs have no change in basal OCR could be due to the presence of increased Akt content (Granlund et al., 2010). Akt, or protein kinase B, is part of a signal transduction pathway responsible for modulating cell growth and proliferation processes. Akt resides inactive in the cytoplasm until activated whereby it migrates to the cell membrane and interacts with PIP3, part of cell receptor signaling cascades. Elevated quantities of Akt may stimulate protein synthesis and therefore is positively correlated with muscle growth and fiber size (Bodine et al., 2001; Schiaffino et al., 2013). Akt could also play a role in regulating mitochondrial homeostasis through downstream effects of mitochondrial redox states, fusion and fission, and energy metabolism (Xie et al., 2022). Beta adrenergic agonists impact the Akt signaling pathway (Lynch and Ryall, 2008; Sato et al., 2011) and thus, may possibly explanation our current basal respiration interaction with isolated mitochondria provided succinate and rotenone. In the current study, control pigs provided BAA can increase mitochondrial respiration through the stimulation of various signaling pathways, however, the AMPKγ3^R200Q^ mutated pigs already have a higher content of Akt and the effects of BAA were not detectable within 1 wk of BAA supplementation. Further research is needed to capture the underlying mechanism of constitutively active AMPK influence on mitochondrial function.

The beta adrenergic agonist ractopamine targets both β_1_ and β_2_ adrenergic receptors (Mills et al., 2003a, 2003b). Curiously, Gunawan et al. (2007) and Gunawan et al. (2020) reported no changes in *β_1_-AR* gene expression but showed decreases in *β_2_-AR* gene expression when pigs were fed 20 ppm RAC for a 4 wk duration. However, in the current study, no differences in *β_2_-AR* gene expression were detected, just a treatment, and treatment by diet interaction in *β_1_-AR* gene expression. This is likely due to a dosage effect on binding to the β_1_-AR receptors at a lower dosage for a shorter duration. Of particular interest is that the increase in *β_1_-AR* expression occurs in the control pig muscle but not in the AMPK γ3^R200Q^ mutant pig muscle. This suggests that constitutively active AMPK γ3^R200Q^ mutant pigs are not responsive to 9 ppm BAA compared to control pigs possibly due to the already upregulated signaling pathways through AMPK, even though ractopamine administration is known to be more effective in oxidative muscles (Gunawan et al., 2020). Given the global impact of constitutively active AMPK on energy metabolism, it is not surprising that modest stimulation by BAA would be over-ridden by a low dose of BAA. Regardless, regulation of beta-adrenoceptors is more complicated in the pigs harboring the AMPKγ3^R200Q^ mutation but certainly warrants further investigation.

## Conclusion

Overall, pigs supplemented with 9 ppm BAA for 1 wk is sufficient to alter muscle metabolism, specifically increases LDH activity, mitochondrial basal respiration, and *β_1_-AR* gene expression. AMPKγ3^R200Q^ mutant pigs have greater muscle oxidative capacity as evident by increases in oxidative enzyme abundance, activity and mitochondrial respiration. Of particular significance, enzyme abundance and activity do not necessarily fluctuate similarly. Specifically, CS activity was lower in isolated mitochondria of AMPKγ3^R200Q^ mutant pig muscle, yet in a whole muscle preparation, CS activity was greater in AMPKγ3^R200Q^ mutant pigs compared to that of control pigs. AMPKγ3^R200Q^ muscle was not as responsive to 9 ppm BAA as that of control pigs suggesting AMPK may impinge on BAA signaling pathways in skeletal muscle. Further investigation is needed regarding the dosage amount, duration of BAA, and potential mechanisms for mitochondria function regulation in AMPKγ3^R200Q^ mutant pigs.

## Abbreviations:

ADP: adenosine diphosphate
AMPK: adenosine monophosphate kinase
BAA: beta adrenergic agonist
CS: citrate synthase
GAPDH: glyceraldehyde 3 phosphate dehydrogenase
LDH: lactate dehydrogenase
OCR: oxygen consumption rate
OXPHOS: oxidative phosphorylation
SDHA: succinate dehydrogenase subunit A
VDAC: voltage dependent anion channel
